# Are Rich People Perceived as More Trustworthy? Perceived Socioeconomic Status Modulates Judgments of Trustworthiness and Trust Behavior Based on Facial Appearance

**DOI:** 10.3389/fpsyg.2018.00512

**Published:** 2018-04-10

**Authors:** Yue Qi, Qi Li, Feng Du

**Affiliations:** ^1^CAS Key Laboratory of Behavioral Science, Institute of Psychology, Chinese Academy of Sciences, Beijing, China; ^2^Department of Psychology, University of Chinese Academy of Sciences, Beijing, China

**Keywords:** socioeconomic status, facial judgment, trust, first impression, cooperation

## Abstract

In the era of globalization, people meet strangers from different countries more often than ever. Previous research indicates that impressions of trustworthiness based on facial appearance play an important role in interpersonal cooperation behaviors. The current study examined whether additional information about socioeconomic status (SES), including national prosperity and individual monthly income, affects facial judgments and appearance-based trust decisions. Besides reproducing previous conclusions that trustworthy faces receive more money than untrustworthy faces, the present study showed that high-income individuals were judged as more trustworthy than low-income individuals, and also were given more money in a trust game. However, trust behaviors were not modulated by the nationality of the faces. The present research suggests that people are more likely to trust strangers with a high income, compared with individuals with a low income.

## Introduction

The information extracted from a stranger’s face (e.g., gender, age, ethnicity and even personality) is critical for forming initial impressions, which play an important role in interpersonal interactions. With a glance of about 33-ms, participants are able to judge the trustworthiness of a stranger’s face (Todorov et al., 2009). A growing body of research indicates that judgments based on facial trustworthiness are crucial for interpersonal trust, which is an important precursor in human cooperation (Zebrowitz and Montepare, 2005, 2008). For example, in a trust game, people tend to invest a larger amount of money in a partner as the imaginary partner’s facial trustworthiness increases (van’t Wout and Sanfey, 2008; Stirrat and Perrett, 2010; Rezlescu et al., 2012). Moreover, people possess a homogeneous ability for forming face-based first impressions across culture and ethnicity, although people treat other-race (socially defined) encounters negatively (Ito and Bartholow, 2009; Ito and Senholzi, 2013). For example, researchers have found that, whether a stranger is from their own or an unfamiliar racial group, people can judge their personality characteristics, such as dominance, from their face (Zebrowitz et al., 1993; Xu et al., 2012). Researchers have also succeeded in predicting presidential election results based on facial competency judgments from different cultures (Sussman et al., 2013). Thus, facial judgment, particularly facial trustworthiness, plays an important role in interpersonal trust and cooperation behaviors.

Trust decisions based on facial appearance can be modulated by some additional information, such as individuals’ previous cooperation behaviors, and personal description of moral character. Past cooperation behaviors influence trustworthiness judgment and trust decisions based on facial appearance. For example, Chang et al. (2010) employed the repeated trust game and found that previous interactions influenced trust behaviors. Here, trust behaviors are defined as how much money was allocated. Furthermore, these initial appraisals of a partner’s facial trustworthiness are modulated not only by the partner’s behavioral histories experienced in previous trust games (Rezlescu et al., 2012), but also by a provided description of reciprocation history (Fareri et al., 2012; Yu et al., 2014). This is also true for information about the moral traits of partners provided before playing trust games, such as from their biographies or information about their recent behavior (Delgado et al., 2005).

Descriptions of individuals’ behavioral history other than general biographies, which indicate their moral qualities, can also modulate trustworthiness judgments based on facial appearance. For example, faces presented with positive descriptions (e.g., “act as a big brother to a fatherless child”) are perceived as being more trustworthy, while faces displayed with negative descriptions (e.g., “engage in unprotected sex after testing positive for HIV”) are perceived as being less trustworthy (Todorov and Olson, 2008; Mende-Siedlecki et al., 2013a). Furthermore, in a trust game, participants were presented with faces of the imaginary partners, along with descriptions of either praiseworthy or suspicious moral character. Participants evaluated the praiseworthy partners as more trustworthy, and thus were more likely to “share” than “keep” (Delgado et al., 2005). These studies illustrate that trust behaviors based on implicit facial impression are also modulated by explicit social background information (e.g., previous cooperation, and information about a partner’s moral character).

Recent studies have shown that social background information other than individuals’ behavioral history and moral character can also influence facial impression. For instance, when we vote for a leader based on his or her face, our preference for a leader’s face is modulated by the environment. Specifically, in war time, people prefer masculine faces, but in peace time, people prefer feminized faces (Little et al., 2007). Similarly, women living in countries with poor healthcare have strong preferences for facial masculinity (measured by facial shape) when making mate choices, and their preference for masculinity increases as the health index of countries decreases (DeBruine et al., 2010). Gender differences had been shown to influence perceived trustworthiness and trust behavior. Specifically, when people act as proposers, men show more trusting behaviors (e.g., giving more money) than women to bolster their own identity (Snijders and Keren, 1999; Chaudhuri and Gangadharan, 2003); when people act as receivers, women show more trustworthy behaviors (e.g., reciprocating more money) than men because women prefer reciprocal exchanges and harmony in the relationship (Croson and Buchan, 1999; Snijders and Keren, 1999; Eckel and Wilson, 2005; Buchan et al., 2008). When it comes to the target’s gender effect on perceived facial trustworthiness, previous results are mixed. Some studies showed trust behavior would not differ by gender of the target (Eckel and Wilson, 2003, 2005; Buchan et al., 2008). In contrast, other research on facial masculinity showed that perceived trustworthiness was associated with target’s gender (Perrett et al., 1998; Johnston et al., 2001; Kruger, 2006; Boothroyd et al., 2007; Macapagal et al., 2011). For example, masculinized faces were judged as less trustworthy (Perrett et al., 1998).

However, few studies have examined whether individual socioeconomic status (SES) can moderate people’s appraisals of another person’s face and corresponding trust behaviors, although there is some indirect evidence. For example, facial width-to-height ratio is positively correlated with aggression among people reporting lower social status or earning lower salaries but not among people reporting high SES (Goetz et al., 2013). Furthermore, there are two kinds of cue that indicate SES can influence people’s attitudes and appraisals. First, the wealth of the nation from which a stranger comes may influence people’s attitude and trust behavior. Based on their impressions on a country, people often assess traits for a typical member of the country (Realo et al., 2009) and even form their attitudes toward the products of the country (O’Shaughnessy and O’Shaughnessy, 2000). The wealth of a nation is a useful index for general well-being for a stranger from another country. Generally speaking, people from high-GDP (gross domestic product per capita) nations have higher subjective well-being and are more trusting and cooperative (Tov and Diener, 2008). Previous studies have also shown that subjective well-being promotes cooperation and prosocial behaviors (Eisenberg, 1991; Thoits and Hewitt, 2001). Naturally, people from high GDP nations are expected to show more cooperative and prosocial behaviors, such as exhibiting more charitable donations (Kraus and Callaghan, 2016). Hence, we expect that people from high GDP nations will be perceived as more cooperative and thus be more trustworthy than people from low GDP nations. Second, individual income also indicates a stranger’s SES. People from high SES families are usually perceived to have better performances and personal traits. For example, students from high-SES families (relatively high household income) were expected to have better academic performance than those from low-SES families (Speybroeck et al., 2012). Several studies have also shown that people from high SES are judged as more favorable, sociable, and attractive (Gilmore and Harris, 2008; Horwitz and Dovidio, 2017). These personal traits are highly correlated with trustworthiness (Oosterhof and Todorov, 2008). Thus, we also expect that people with high income are perceived as more trustworthy than people with low income.

The present study was designed to examine whether two types of SES information, national prosperity and monthly income, can modulate facial trustworthiness judgment and subsequent trust behavior. In addition, we also explored whether trust behavior based on facial appearance is influenced by the race of a face. Recent studies showed that the trustworthiness evaluations rely on the face shape, not ethnicity. Actually, own-race faces were rated as trustworthy as other-race faces (Xu et al., 2012; Birkás et al., 2014; Sofer et al., 2017; Strachan et al., 2017). Therefore, we expected that the two types of SES information would modulate facial judgment and trust behavior, but race would not. Specifically, those faces paired with higher SES would be perceived as more trustworthy than the faces paired with lower SES.

## Experiment 1

This experiment used a trust game task to examine whether people’s trust behaviors based on a partner’s facial trustworthiness would be modulated by the nationality of the partner.

### Methods

#### Participants

A group of 32 undergraduate and graduate students (16 males) participated in a half-hour-long experiment and were offered 30 yuan (∼$4.29) as compensation for their time. Each participant provided written informed consent before the experiment. The study was approved by the Institutional Review Board of the Institute of Psychology, Chinese Academy of Sciences.

#### Stimuli

67 face pictures were selected from the FERET (Phillips et al., 1998, 2000) and CAS-PEAL (Gao et al., 2008) databases, including 35 Asian faces (17 males) and 32 Caucasian faces (16 males). Facial trustworthiness was rated on a 9-point scale by a separate group of 20 subjects, and then divided into two groups as faces with high trustworthiness (mean ratings for Asian faces was 5.66 ± 0.58; Caucasian faces 6.00 ± 0.45) and faces with low trustworthiness (mean ratings for Asian faces was 4.13 ± 0.52; Caucasian faces 3.68 ± 0.50). Considering people have an inaccurate stereotype for their own country (Robins, 2005), four countries were selected from the World Bank report in 2013, including high- and low-GDP countries in Asia and Europe, excluding China (see **Table 1**). Another 54 subjects rated how much they were familiar with each country on a 5-point Likert scale. The familiarity ratings of Singapore compared with Vietnam [*t*(53) = 1.84, *p* = 0.071], and Norway compared with Ukraine [*t*(53) = 0.47, *p* = 0.642], showed no differences.

**Table 1:** Information about the four countries used in Experiment 1.

	Singapore	Vietnam	Norway	Ukraine
Per capita GDP	$55182	$1911	$100819	$3900
Rank^†^	12	137	4	110
Familiarity	3.31 ± 0.91	3.15 ± 0.98	2.89 ± 0.88	2.93 ± 0.93

*^†^Data from the World Bank report in 2013.*

#### Apparatus and Procedure

**FIGURE 1:**
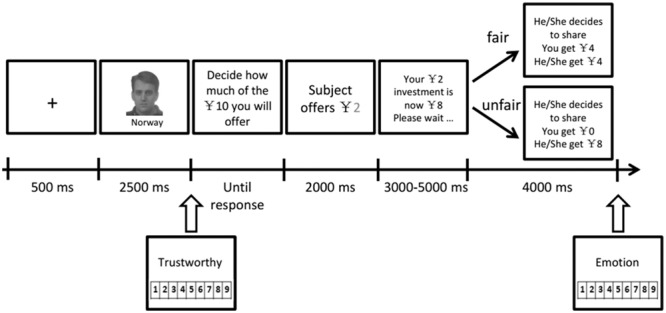
Experimental design with an example of the sequence and timing of stimuli in a typical trial.

Participants were told that they were playing an online money distribution game with some other people. Before the experiment, every participant posed for a picture with a neutral expression and was told that their photograph would be seen by their partners during the experiment. All participants were seated comfortably in a dimly lit and sound-attenuating chamber approximately 80 cm away from a computer screen. A single trial consisted of the following sequence: initially, a face photo with a country name was displayed in the center of the screen for 2500 ms. Then, participants were required to give a trustworthiness rating for that facial photo. After that, participants were asked to decide how much of the 10 yuan (from 1 to 10 yuan) they would give to this partner in this trial. Their partner would receive quadruple the amount of money allocated by the participant and distribute it either fairly (5:5) or unfairly (3:1 or the partner keeps all the money). Participants would see the distribution results at the end of each trial and were instructed to evaluate their current emotion on a 9-point scale, where 1 indicated sad and 9 indicated happy (see **Figure 1**).

The task consisted of 67 trials divided into two blocks, with a short break between blocks. A practice block with eight trials was completed before the formal experiment in order to familiarize the participants with the procedure. Before the formal experiment, the participants were encouraged to use any strategy they wanted to maximize their amount of points. At the end of the experiment, one trial would be selected randomly and the actual result of the trial would be the bonus money that participant would receive.

#### Design

There were two independent variables of interest: national prosperity (high- vs. low-GDP countries) and facial trustworthiness (high vs. low trustworthiness). The combinations of country names and faces were counterbalanced between subjects, to ensure every face appeared with both a high-GDP and a low-GDP country name.

### Results

#### Trust-Rating

First, to explore whether the nationalities of partners would influence perceived facial trustworthiness, we compared the trust judgments on the same face displayed with high- or low-GDP countries. *T*-test analysis revealed that there were no differences between faces with high and low GDP countries, either for Asian [*t*(34) = 1.63, *p* = 0.113] or for Caucasian faces [*t*(31) = 1.18, *p* = 0.247]. Participants judged facial trustworthiness without the influence of nationality.

#### Money

**FIGURE 2:**
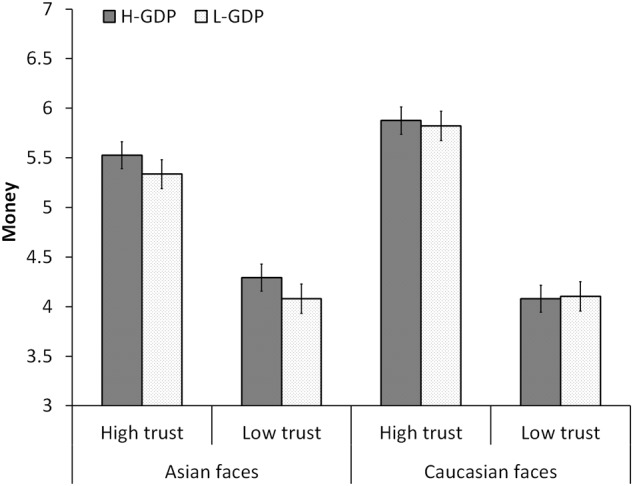
Mean money distributed to partners in Experiment 1. “H-GDP” means the countries with a higher GDP rank, such as Singapore and Norway; “L-GDP” means the countries with a lower GDP rank, such as Vietnam and Ukraine. Error bars indicate the SEM.

The mean money distributed to partners is shown in **Figure 2**. First, a 2 (national prosperity) × 2 (facial trustworthiness) × 2 (facial race) ANOVA revealed the main effect of facial trustworthiness, *F*(1, 31) = 99.78, *p* < 0.001, $\eta_{p}^{2}$ = 0.763. Participants allocated more money to high-trustworthy faces (5.64 ± 0.29) than to low-trustworthy faces (4.14 ± 0.28). There was neither a significant main effect of national prosperity, *F*(1, 31) = 1.55, *p* = 0.223, $\eta_{p}^{2}$ = 0.048, nor the main effect of race, *F*(1, 31) = 2.57, *p* = 0.119, $\eta_{p}^{2}$ = 0.077. There was a significant interaction of facial race and trustworthiness^1^, *F*(1, 31) = 9.21, *p* = 0.005, $\eta_{p}^{2}$ = 0.229. The three-way interaction [*F*(1, 31) = 1.00, *p* = 0.755, $\eta_{p}^{2}$ = 0.003] or the interactions between national prosperity and facial trustworthy [*F*(1, 31) = 0.05, *p* = 0.825, $\eta_{p}^{2}$= 0.002], between race and national prosperity [*F*(1, 31) = 1.64, *p* = 0.210, $\eta_{p}^{2}$ = 0.050] were not significant. Although participants showed more trust behavior toward the high-trustworthy faces, their trust judgments were not modulated by the national prosperity of the partners.

A 2 (facial gender) × 2 (participant’s gender) ANOVA was conducted to analyze gender effects on allocated money. The results revealed the main effect of participant’s gender, *F*(1, 30) = 6.60, *p* = 0.015, $\eta_{p}^{2}$ = 0.180. Male participants (5.59 ± 0.36) sent more money than female participants (4.27 ± 0.36). The main effect of facial gender [*F*(1, 30) = 0.12, *p* = 0.727, $\eta_{p}^{2}$ = 0.004] or the interaction between facial and participant’s gender [*F*(1, 30) = 3.43, *p* = 0.074, $\eta_{p}^{2}$ = 0.102] were not significant.

#### Emotion

A 2 (national prosperity) × 2 (facial race) × 2 (fair/unfair conditions) ANOVA showed a significant main effect of the distribution results, *F*(1, 31) = 93.60, *p* < 0.001, $\eta_{p}^{2}$ = 0.751. People felt more negative after seeing unfair distributions (3.36 ± 0.19) than fair distributions (6.35 ± 0.19). The main effects of national prosperity [*F*(1, 31) = 0.77, *p* = 0.388, $\eta_{p}^{2}$ = 0.024] and race [*F*(1, 31) = 0.50, *p* = 0.824, $\eta_{p}^{2}$ = 0.002] were not significant. The three-way interaction [*F*(1, 31) = 0.06, *p* = 0.811, $\eta_{p}^{2}$ = 0.002] or any two-way interaction [*F*_fair × nationality_ (1, 31) = 0.66, *p* = 0.424, $\eta_{p}^{2}$ = 0.021; F_fair × race_ (1, 31) = 1.20, *p* = 0.282, $\eta_{p}^{2}$ = 0.037; *F*_race × nationality_ (1, 31) = 0.44, *p* = 0.512, $\eta_{p}^{2}$ = 0.014] were not significant, which indicted that participants’ emotions were affected only by money distribution results but not the partner’s race or nationality.

## Experiment 2

Experiment 1 verified that the partner’s facial trustworthiness affects people’s trust behaviors. However, the nationalities of the partners did not modulate people’s face-based trust behaviors. Experiment 2 examined whether people’s trust behaviors based on the partner’s facial trustworthiness would be modulated by the partner’s financial situation such as monthly income.

### Methods

#### Participants

Another 33 undergraduate and graduate students (16 males) participated in this experiment. They gave signed informed consent and received cash compensation for their time.

#### Stimuli, Apparatus, and Procedure

Experiment 2 was identical to Experiment 1, with one exception. Participants were instructed to report their monthly income after their photo was taken. Then, at the beginning of each trial, a face photo with a monthly income was displayed in the center of the screen. Since all participants were students, we selected 2000/lower yuan (∼$286) per month as low income, and 10001/higher yuan (∼$2857) as high income.

### Results

#### Trust-Rating

First, to explore whether the monthly income of partners influences perceived facial trustworthiness, we compared the trust judgments on the same face paired with high or low income. *T*-test analysis revealed that there was no difference in perceived facial trustworthiness for high and low monthly income for Asian faces [*t*(34) = 1.33, *p* = 0.193]. However, for Caucasian faces, those paired with higher income were rated higher for trustworthiness than were faces paired with lower income [*t*(31) = 3.39, *p* = 0.002]. Perceived facial trustworthiness was affected by personal income for other-race faces.

#### Money

**FIGURE 3:**
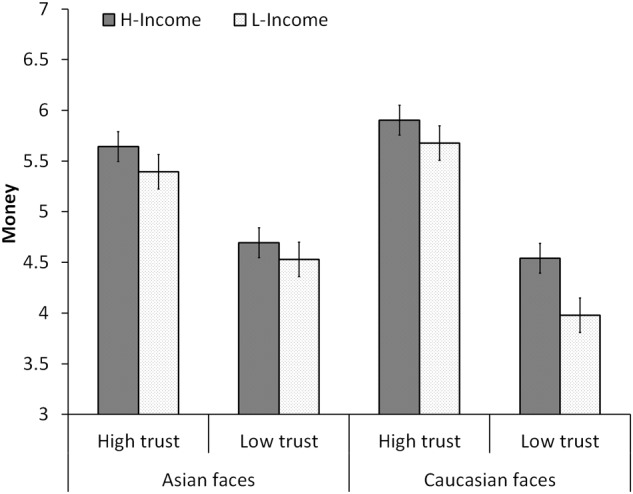
Mean money distributed to partners in Experiment 2. “H-Income” means higher monthly income; “L-Income” means lower monthly income. Error bars indicate the SEM.

The mean money distributed to partners is shown in **Figure 3**. First, a 2 (income) × 2 (facial trustworthiness) × 2 (facial race) ANOVA revealed the main effect of facial trustworthiness, *F*(1, 32) = 55.10, *p* < 0.001, $\eta_{p}^{2}$ = 0.633. Participants allocated more money to high-trustworthy faces (5.65 ± 0.40) than to low-trustworthy faces (4.43 ± 0.38). We also found a main effect of personal income, *F*(1, 32) = 5.96, *p* = 0.035, $\eta_{p}^{2}$ = 0.132. Participants allocated more money to high-income ones (5.19 ± 0.39) than to low-income ones (4.89 ± 0.39). The main effect of race was not significant, *F*(1, 32) = 0.11, *p* = 0.737, $\eta_{p}^{2}$ = 0.004. There was a significant interaction of facial race and trustworthiness^1^ [*F*(1, 32) = 11.17, *p* = 0.002, $\eta_{p}^{2}$ = 0.259]. The three-way interaction [*F*(1, 32) = 2.46, *p* = 0.127, $\eta_{p}^{2}$ = 0.071] or the interactions between income and facial trustworthiness [*F*(1, 32) = 0.64, *p* = 0.430, $\eta_{p}^{2}$ = 0.020], between race and income [*F*(1, 32) = 2.38, *p* = 0.133, $\eta_{p}^{2}$ = 0.069] were not significant.

A 2 (facial gender) × 2 (participant’s gender) ANOVA was conducted to analyze gender effects on allocated money. The results revealed the main effect of participant’s gender, *F*(1, 31) = 8.50, *p* = 0.007, $\eta_{p}^{2}$ = 0.215. Male participants (6.11 ± 0.49) sent more money than female participants (4.12 ± 0.48). The main effect of facial gender [*F*(1, 31) = 0.11, *p* = 0.744, $\eta_{p}^{2}$ = 0.003] or the interaction between facial and participant’s gender [*F*(1, 31) = 3.67, *p* = 0.065, $\eta_{p}^{2}$ = 0.106] were not significant.

#### Emotion

A 2 (income) × 2 (facial race) × 2 (fair/unfair conditions) ANOVA revealed a significant main effect of the distribution results, *F*(1, 32) = 88.99, *p* < 0.001, $\eta_{p}^{2}$ = 0.736. People felt more negative after seeing unfair distributions (3.47 ± 0.20) than fair distributions (6.39 ± 0.18). The main effects of income [*F*(1, 32) = 0.59, *p* = 0.447, $\eta_{p}^{2}$ = 0.018] and race [*F*(1, 32) = 0.48, *p* = 0.492, $\eta_{p}^{2}$ = 0.015] were not significant. The three-way interaction [*F*(1, 32) = 0.50, *p* = 0.486, $\eta_{p}^{2}$ = 0.015], the interaction between race and distribution condition [*F*(1, 32) = 2.52, *p* = 0.122, $\eta_{p}^{2}$ = 0.073] or between race and income [*F*(1, 32) = 1.26, *p* = 0.270, $\eta_{p}^{2}$ = 0.038] were not significant. However, the interaction between distribution condition and partner’s monthly income was significant, *F*(1, 32) = 8.41, *p* = 0.007, $\eta_{p}^{2}$ = 0.208. Further multiple comparisons with Bonferroni correction showed that when the distributions were fair, participants felt more positive emotions when the partner had a high income than a low income (*p* = 0.017); but when the distributions were unfair, participants’ emotions were not modulated by the partner’s income (*p* = 0.129).

## Discussion

The present study showed that an individual’s monthly income can modulate trustworthiness judgments and subsequent trust behavior based on facial appearance. Thus the present study is the first to show that information about an individual’s monthly income rather than the wealth associated with his/her nationality can influence perceived facial trustworthiness and subsequent trust behavior. This indicates an important role for personal economic status in shaping the trustworthiness judgment based on a stranger’s face.

There are two possible explanations for the effect of individual income on trustworthiness judgment and actual trust behaviors based on facial appearance in Experiment 2. Firstly, many previous studies have shown that an individual’s ability is a key factor in shaping perceived trustworthiness (Mayer et al., 1995). Since an individual’s income is an index of that individual’s ability (Judge et al., 2009), the information about an individual’s income might also influence perceived facial trustworthiness and subsequent trust decisions. As a result, the imaginary partners who were paired with higher income would be perceived as more capable and more trustworthy than partners with lower income. Through manipulating high- or low-SES background information, a previous study demonstrated that low-SES students were perceived as having less positive personal characteristics, greater need for academic support, and less promising futures than other students; additionally, teachers felt ineffective when working with students with a lower social status (Auwarter and Aruguete, 2008). Moreover, a meta-analysis of studies on mock juror judgments showed that low SES defendants were found guilty more easily and received greater punishment for their crimes than did others, indicating jurors’ bias against low SES defendants (Mazzella and Feingold, 1994). This finding was also verified in a recent study (Farnum and Stevenson, 2013). In addition to explicit prejudice against low-SES people, participants also express implicit pro-rich attitudes (Horwitz and Dovidio, 2017). Although these results are not directly tested in facial judgment, these findings show that an individual’s SES can influence their perceived ability, which might further moderate perceived trustworthiness. The present findings give further direct evidence that monthly income, as an index of an individual’s capability, affects perceived face-based trustworthiness and trust behavior.

However, perceived trustworthiness also depends on speculation about a person’s motivation to cooperate or betray. People tend to trust those partners who are perceived as having positive intentions toward others (Lount and Pettit, 2012). High-income individuals are usually more generous, and thus perceived as being more likely to cooperate in such a money exchange game than poor people, because the money obtained in the experiment is insignificant for them (Ermisch and Gambetta, 2011). Furthermore, previous evidence demonstrates that increases in individual income predict corresponding increases in social trust, generosity and charity behavior (Ermisch and Gambetta, 2011; Brandt et al., 2015; Shaleva, 2015). This is partly because people with a higher SES have more resources to tolerate the risks of trust than do other people (Hamamura, 2012). Once a stereotype that rich people are more generous has been formed, people tend to perceive rich people as more trustworthy than others from a lower SES.

Surprisingly, in Experiment 1, we found no effect of nationality on facial trustworthiness judgment or trust behavior. One possibility is that, just as people’s perceptions of national characters may not be generalized to the assessment of a specific person’s traits (Terracciano et al., 2005), the stereotypes about the wealth of a country may not be generalized to the inferences about individual trustworthiness. Another reason is that perceived individual trustworthiness based on facial appearance is different from an individual’s tendency to trust in others. Although people from wealthy countries might be more likely to trust in others, the wealth of a person’s home country may not modulate face-based trustworthiness judgment and trust (Eisenberg, 1991; Thoits and Hewitt, 2001). Unlike individual monthly income, the participant might not spontaneously link national prosperity with individual SES. Future study with an explicit instruction to connect national prosperity with individual SES might be needed.

From the results of the current studies, some, but not all, additional information will influence appearance-based trust behavior. This is consistent with previous studies showing that the additional information can facilitate the updating of impressions based on facial cues (Singer et al., 2004; Delgado et al., 2005; Mende-Siedlecki et al., 2013b). However, this may only happen when people are given individual information which directly relates to a stranger’s social status, not indirect information related to group or nation.

Moreover, previous studies have shown that the higher facial trustworthiness for same-race faces can boost viewers’ trust behaviors (DeBruine, 2002; van’t Wout and Sanfey, 2008). The present results expand on this finding by showing the effect of facial trustworthiness on people’s trust behavior for both same- and other-race faces. Though some previous studies found other-race partners were perceived as more risky than were same-race partners (Blair et al., 2004; Eberhardt et al., 2006), our results are consistent with previous findings of cross-cultural homogeneity regarding facial judgments (Mcarthur and Berry, 1987; Zebrowitz et al., 1993; Birkás et al., 2014). For instance, an online trust game among Arab and German participants relied on similar facial features to judge trustworthiness cross culture (Bente et al., 2014).

Consistent with previous research (Snijders and Keren, 1999; Chaudhuri and Gangadharan, 2003), male participants showed more trusting behaviors and sent more money to their counterparts than female participants in both experiments. As the social role theory pointed out, differences in social behaviors depend on gender roles, which is associated with expectancies and acquired skills and beliefs (Eagly and Wood, 1991; Eagly, 1997). Specifically, men are more instrumental and aggressive whereas women tend to be more social and empathetic (Bakan, 1966; Anderson and Blanchard, 1982; Eagly and Steffen, 1986; Ickes et al., 1986). To build good reputation among strangers, male participants show more trusting behaviors to other people.

In our current studies, participants were told that they would actually earn the money based on their decisions. Actual money could help to convince participants that they were playing this trust game with real people, and motivate serious investment choices (e.g., Bohnet and Zeckhauser, 2004; Kosfeld et al., 2005; Rezlescu et al., 2012; Bente et al., 2014). Furthermore, previous research confirms that there is no difference between actual and simulated money (e.g., Johnson and Bickel, 2002; Madden et al., 2003; Lagorio and Madden, 2005). For example, by using a within-subject design, no systematic difference was found in response to real and hypothetical rewards (Johnson and Bickel, 2002). Hence, the similar results can be expected by using simulated money.

Further research is necessary to consolidate these findings and to better explain the observed results. An important question for further study is whether this trust preference for richer people is only shown by relatively low income groups, such as graduate and undergraduate students. Moreover, using relative performance ranks in a temporal estimation task, people showed a lower tendency to accept unfair offers from a high status partner compared with a low status partner (Hu et al., 2014). Thus, what kind of information indicating relative status would influence face-based perceived trustworthiness, and whether it is modulated by people’s stereotypes, are further questions to be answered. Additionally, though sample sizes in the current studies were decided on the basis of similar research on social judgments of face (Rogers et al., 2014; Manssuer et al., 2016; Strachan et al., 2016, 2017; Sofer et al., 2017; Strachan and Tipper, 2017), the sample size was lower than the recommendations of Fraley and Vazire’s study (Fraley and Vazire, 2014). Future research with a larger sample size might be needed.

In summary, the current research showed that people tend to trust strangers with trustworthy faces from a higher SES compared with individuals with untrustworthy faces or from a lower SES. Therefore, the current studies indicate that an individual’s economic status is not only related to their health and life satisfaction, but also to their perceived trustworthiness. People should be aware of this bias toward higher SES during impression formation, especially when they are going to make some crucial cooperative decisions. Further research is necessary to explore whether this trust preference for higher SES is only demonstrated by relatively low income groups, such as graduate and undergraduate students.

## Author Contributions

YQ and FD conceived and designed the study and wrote the paper. YQ and QL carried out the literature search and synthesis.

## Conflict of Interest Statement

The authors declare that the research was conducted in the absence of any commercial or financial relationships that could be construed as a potential conflict of interest.

## Footnotes

1 We speculated that the interaction of race and facial trustworthiness was caused by the different original trust-rating for Asian and Caucasian face photos. The original facial trustworthiness ratings were 5.66 ± 0.61 (high-trustworthiness) and 4.13 ± 1.02 (low-trustworthiness) for Asian faces; 6.00 ± 0.80 (high-trustworthiness) and 3.68 ± 0.90 (low-trustworthiness) for Caucasian faces. The original rating difference between high- and low-trustworthy face photos was smaller for Asian faces than it for Caucasian faces [*F*(1, 19) = 18.09, *p* < 0.001, $\eta_{p}^{2}$ = 0.488]. Hence, the differences of allocated money between high- and low-trustworthy face photos were also smaller for Asian faces than it for Caucasian faces, which caused the interaction here. Future studies with better balanced face photos might be needed.

## References

[B1] Anderson L. Blanchard N. (1982). Sex differences in task and social-emotional behavior. *Basic Appl. Soc. Psychol.* 3 109–139. 10.1207/s15324834basp0302_3

[B2] Auwarter A. E. Aruguete M. S. (2008). Effects of student gender and socioeconomic status on teacher perceptions. *J. Educ. Res.* 101 242–246. 10.3200/JOER.101.4.243-246

[B3] Bakan D. (1966). *The Duality of Human Existence: An Essay on Psychology and Religion.* Chicago, IL: Rand McNally.

[B4] Bente G. Dratsch T. Kaspar K. Häßler T. Bungard O. Al-Issa A. (2014). Cultures of trust: effects of avatar faces and reputation scores on German and Arab players in an online trust-game. *PLoS One* 9:e98297. 10.1371/journal.pone.0098297 24901696 PMC4046985

[B5] Birkás B. Dzhelyova M. Lábadi B. Bereczkei T. Perrett D. I. (2014). Cross-cultural perception of trustworthiness: the effect of ethnicity features on evaluation of faces’ observed trustworthiness across four samples. *Pers. Individ. Dif.* 69 56–61. 10.1016/j.paid.2014.05.012

[B6] Blair I. V. Judd C. M. Chapleau K. M. (2004). The influence of Afrocentric facial features in criminal sentencing. *Psychol. Sci.* 15 674–679. 10.1111/j.0956-7976.2004.00739.x 15447638

[B7] Bohnet I. Zeckhauser R. (2004). Social comparisons in ultimatum bargaining. *Scand. J. Econ.* 106 495–510. 10.1111/j.0347-0520.2004.00376.x

[B8] Boothroyd L. G. Jones B. C. Burt D. M. Perrett D. I. (2007). Partner characteristics associated with masculinity, health, and maturity in male faces. *Pers. Individ. Dif.* 43 1161–1173. 10.1016/j.paid.2007.03.008

[B9] Brandt M. J. Wetherell G. Henry P. J. (2015). Changes in income predict change in social trust: a longitudinal analysis. *Polit. Psychol.* 36 761–768. 10.1111/pops.12228

[B10] Buchan N. R. Croson R. T. A. Solnick S. (2008). Trust and gender: an examination of behavior and beliefs in the investment game. *J. Econ. Behav. Organ.* 68 466–476. 10.1016/j.jebo.2007.10.006

[B11] Chang L. J. Doll B. B. van ’t Wout M. Frank M. J. Sanfey A. G. (2010). Seeing is believing: trustworthiness as a dynamic belief. *Cogn. Psychol.* 61 87–105. 10.1016/j.cogpsych.2010.03.001 20553763

[B12] Chaudhuri A. Gangadharan L. (2003). *Gender Differences in Trust and Reciprocity.* Auckland: The University of Auckland 248.

[B13] Croson R. Buchan N. (1999). Gender and culture: international experimental evidence from trust games. *Am. Econ. Rev.* 89 386–391. 10.1257/aer.89.2.386

[B14] DeBruine L. M. (2002). Facial resemblance enhances trust. *Proc. R. Soc. B Biol. Sci.* 269 1307–1312. 10.1098/rspb.2002.2034 12079651 PMC1691034

[B15] DeBruine L. M. Jones B. C. Crawford J. R. Welling L. L. M. Little A. C. (2010). The health of a nation predicts their mate preferences: cross-cultural variation in women’s preferences for masculinized male faces. *Proc. R. Soc. B Biol. Sci.* 277 2405–2410. 10.1098/rspb.2009.2184 20236978 PMC2894896

[B16] Delgado M. R. Frank R. H. Phelps E. A. (2005). Perceptions of moral character modulate the neural systems of reward during the trust game. *Nat. Neurosci.* 8 1611–1618. 10.1038/nn1575 16222226

[B17] Eagly A. (1997). Sex differences in social behavior: comparing social role theory and evolutionary psychology. *Am. Psychol.* 52 1380–1383. 10.1037/0003-066X.52.12.1380.b 9414607

[B18] Eagly A. Steffen V. (1986). Gender and aggressive behavior: a meta-analytic review of the social psychological literature. *Psychol. Bull.* 100 309–330. 10.1037/0033-2909.100.3.309 3797558

[B19] Eagly A. Wood W. (1991). Explaining sex differences in social behavior: a meta-analytic perspective. *Pers. Soc. Psychol. Bull.* 17 306–315. 10.1177/0146167291173011

[B20] Eberhardt J. L. Davies P. G. Purdie-Vaughns V. J. Johnson S. L. (2006). Looking deathworthy: perceived stereotypicality of black defendants predicts capital-sentencing outcomes. *Psychol. Sci.* 17 383–386. 10.1111/j.1467-9280.2006.01716.x 16683924

[B21] Eckel C. Wilson R. (2003). *Conditional Trust: Sex, Race and Facial Expressions in an Experimental Trust Game.* Virginia, NV: Virginia Tech University.

[B22] Eckel C. Wilson R. (2005). *Attractiveness and Trust: Does Beauty Confound Intuition*? Virginia, NV: Virginia Tech University.

[B23] Eisenberg N. (1991). Meta-analytic contributions to the literature on prosocial behavior. *Pers. Soc. Psychol. Bull.* 17 273–282. 10.1177/0146167291173007

[B24] Ermisch J. Gambetta D. (2011). *The Long Shadow of Income on Trustworthiness. IZA Discussion Paper, No. 5585.* Available at: https://ssrn.com/abstract=1796540

[B25] Fareri D. S. Chang L. J. Delgado M. R. (2012). Effects of direct social experience on trust decisions and neural reward circuitry. *Front. Neurosci.* 6:148. 10.3389/fnins.2012.00148 23087604 PMC3472892

[B26] Farnum K. S. Stevenson M. C. (2013). Economically disadvantaged juvenile offenders tried in adult court are perceived as less able to understand their actions, but more guilty. *Psychol. Crime Law* 19 727–744. 10.1080/1068316X.2013.793766

[B27] Fraley R. C. Vazire S. (2014). The N-pact factor: evaluating the quality of empirical journals with respect to sample size and statistical power. *PLoS One* 9:e109019. 10.1371/journal.pone.0109019 25296159 PMC4189949

[B28] Gao W. Cao B. Shan S. G. Chen X. L. Zhou D. L. Zhang X. H. (2008). The CAS-PEAL large-scale Chinese face database and baseline evaluations. *IEEE Trans. Syst. Man Cybern. Part A Syst. Hum.* 38 149–161. 10.1109/Tsmca.2007.909557

[B29] Gilmore A. K. Harris P. B. (2008). Socioeconomic stereotypes among undergraduate college students. *Psychol. Rep.* 103 882–892. 10.2466/pr0.103.3.882-892 19320225

[B30] Goetz S. M. M. Shattuck K. S. Miller R. M. Campbell J. A. Lozoya E. Weisfeld G. E. (2013). Social status moderates the relationship between facial structure and aggression. *Psychol. Sci.* 24 2329–2334. 10.1177/0956797613493294 24068116

[B31] Hamamura T. (2012). Social class predicts generalized trust but only in wealthy societies. *J. Cross Cult. Psychol.* 43 498–509. 10.1177/0022022111399649

[B32] Horwitz S. R. Dovidio J. F. (2017). The rich—love them or hate them? Divergent implicit and explicit attitudes toward the wealthy. *Group Process. Intergroup Relat.* 20 3–31. 10.1177/1368430215596075

[B33] Hu J. Cao Y. Blue P. R. Zhou X. (2014). Low social status decreases the neural salience of unfairness. *Front. Behav. Neurosci.* 8:402. 10.3389/fnbeh.2014.00402 25477798 PMC4238404

[B34] Ickes W. Robertson E. Tooke W. Teng G. (1986). Naturalistic social cognition: methodology, assessment, and validation. *J. Pers. Soc. Psychol.* 51 66–82. 10.1037/0022-3514.51.1.66

[B35] Ito T. A. Bartholow B. D. (2009). The neural correlates of race. *Trends Cogn. Sci.* 13 524–531. 10.1016/j.tics.2009.10.002 19896410 PMC2796452

[B36] Ito T. A. Senholzi K. B. (2013). Us versus them: understanding the process of race perception with event-related brain potentials. *Vis. Cogn.* 21 1096–1120. 10.1080/13506285.2013.821430

[B37] Johnson M. W. Bickel W. K. (2002). Within-subject comparison of real and hypothetical money rewards in delay discounting. *J. Exp. Anal. Behav.* 77 129–146. 10.1901/jeab.2002.77-129 11936247 PMC1284852

[B38] Johnston V. Hagel R. Franklin M. Fink B. Grammer K. (2001). Male facial attractiveness: evidence for hormone-related adaptive design. *Evol. Hum. Behav.* 22 251–267. 10.1016/S1090-5138(01)00066-6

[B39] Judge T. A. Hurst C. Simon L. S. (2009). Does it pay to be smart, attractive, or confident (or all three)? Relationships among general mental ability, physical attractiveness, core self-evaluations, and income. *J. Appl. Psychol.* 94 742–755. 10.1037/a0015497 19450010

[B40] Kosfeld M. Heinrichs M. Zak P. J. Fischbacher U. Fehr E. (2005). Oxytocin increases trust in humans. *Nature* 435 673–676. 10.1038/nature03701 15931222

[B41] Kraus M. W. Callaghan B. (2016). Social class and prosocial behavior: the moderating role of public versus private contexts. *Soc. Psychol. Pers. Sci.* 7 769–777. 10.1177/1948550616659120

[B42] Kruger D. J. (2006). Male facial masculinity influences attributions of personality and reproductive strategy. *Pers. Relationsh.* 13 451–463. 10.1111/j.1475-6811.2006.00129.x

[B43] Lagorio C. H. Madden G. J. (2005). Delay discounting of real and hypothetical rewards III: steady-state assessments, forced-choice trials, and all real rewards. *Behav. Process.* 69 173–187. 10.1016/j.beproc.2005.02.003 15845306

[B44] Little A. C. Burriss R. P. Jones B. C. Roberts S. C. (2007). Facial appearance affects voting decisions. *Evol. Hum. Behav.* 28 18–27. 10.1016/j.evolhumbehav.2006.09.002

[B45] Lount R. B. Pettit N. C. (2012). The social context of trust: the role of status. *Organ. Behav. Hum. Decis. Process.* 117 15–23. 10.1016/j.obhdp.2011.07.005.

[B46] Macapagal K. R. Rupp H. A. Heiman J. R. (2011). Influences of observer sex, facial masculinity, and gender role identification on first impressions of men’s faces. *J. Soc. Evol. Cult. Psychol.* 5 92–105. 10.1037/h0099273 21874151 PMC3162194

[B47] Madden G. J. Begotka A. M. Raiff B. R. Kastern L. L. (2003). Delay discounting of real and hypothetical rewards. *Exp. Clin. Psychopharmacol.* 11 139–145. 10.1037/1064-1297.11.2.139 12755458

[B48] Manssuer L. R. Pawling R. Hayes A. E. Tipper S. P. (2016). The role of emotion in learning trustworthiness from eye-gaze: evidence from facial electromyography. *Cogn. Neurosci.* 7 82–102. 10.1080/17588928.2015.1085374 27153239 PMC4867790

[B49] Mayer R. C. Davis J. H. Schoorman F. D. (1995). An integrative model of organizational trust. *Acad. Manage. Rev.* 20 709–734. 10.5465/amr.1995.9508080335

[B50] Mazzella R. Feingold A. (1994). The effects of physical attractiveness, race, socioeconomic status, and gender of defendants and victims on judgments of mock jurors: a meta-analysis. *J. Appl. Soc. Psychol.* 24 1315–1338. 10.1111/j.1559-1816.1994.tb01552.x

[B51] Mcarthur L. Z. Berry D. S. (1987). Cross-cultural agreement in perceptions of babyfaced adults. *J. Cross Cult. Psychol.* 18 165–192. 10.1177/0022002187018002003

[B52] Mende-Siedlecki P. Cai Y. Todorov A. (2013a). The neural dynamics of updating person impressions. *Soc. Cogn. Affect. Neurosci.* 8 623–631. 10.1093/Scan/Nss040 22490923 PMC3739907

[B53] Mende-Siedlecki P. Said C. P. Todorov A. (2013b). The social evaluation of faces: a meta-analysis of functional neuroimaging studies. *Soc. Cogn. Affect. Neurosci.* 8 285–299. 10.1093/scan/nsr090 22287188 PMC3594716

[B54] Oosterhof N. N. Todorov A. (2008). The functional basis of face evaluation. *Proc. Natl. Acad. Sci. U.S.A.* 105 11087–11092. 10.1073/pnas.0805664105 18685089 PMC2516255

[B55] O’Shaughnessy J. O’Shaughnessy N. J. (2000). Treating the nation as a brand: some neglected issues. *J. Macromark.* 20 56–64. 10.1177/0276146700201006

[B56] Perrett D. I. Lee K. J. Penton-Voak I. Rowland D. Yoshikawa S. Burt D. (1998). Effects of sexual dimorphism on facial attractiveness. *Nature* 394 884–887. 10.1038/29772 9732869

[B57] Phillips P. J. Moon H. Rizvi S. A. Rauss P. J. (2000). The FERET evaluation methodology for face-recognition algorithms. *IEEE Trans. Pattern Anal. Mach. Intell.* 22 1090–1104. 10.1109/34.879790

[B58] Phillips P. J. Wechsler H. Huang J. Rauss P. J. (1998). The FERET database and evaluation procedure for face-recognition algorithms. *Image Vis. Comput.* 16 295–306. 10.1016/S0262-8856(97)00070-X

[B59] Realo A. Allik J. Lönnqvist J.-E. Verkasalo M. Kwiatkowska A. Kööts L. (2009). Mechanisms of the national character stereotype: how people in six neighbouring countries of Russia describe themselves and the typical Russian. *Eur. J. Pers.* 23 229–249. 10.1002/per.719

[B60] Rezlescu C. Duchaine B. Olivola C. Y. Chater N. (2012). Unfakeable facial configurations affect strategic choices in trust games with or without information about past behavior. *PLoS One* 7:e34293. 10.1371/journal.pone.0034293 22470553 PMC3314625

[B61] Robins R. W. (2005). The nature of personality: genes, culture, and national character. *Science* 310 62–63. 10.1126/science.1119736 16210523

[B62] Rogers R. D. Bayliss A. P. Szepietowska A. Dale L. Reeder L. Pizzamiglio G. Tipper S. P. (2014). I want to help you, but I am not sure why: gaze-cuing induces altruistic giving. *J. Exp. Psychol.* 143 763–777. 10.1037/a0033677 23937180 PMC3970851

[B63] Shaleva A. E. (2015). Uncovering the impact of intergenerational income mobility on interpersonal trust. *IZA J. Labor Dev.* 4:5 10.1186/s40175-015-0037-3

[B64] Singer T. Kiebel S. J. Winston J. S. Dolan R. J. Frith C. D. (2004). Brain responses to the acquired moral status of faces. *Neuron* 41 653–662. 10.1016/S0896-6273(04)00014-5 14980212

[B65] Snijders C. Keren G. (1999). “Determinants of trust”, in *Games and Human Behavior* eds Budescu D. Erev I. Zwick R. (Mahwah, NJ: Lawrence Erlbaum Associates) 355–385.

[B66] Sofer C. Dotsch R. Oikawa M. Oikawa H. Wigboldus D. H. J. Todorov A. (2017). For your local eyes only: culture-specific face typicality influences perceptions of trustworthiness. *Perception* 46 914–928. 10.1177/0301006617691786 28152651

[B67] Speybroeck S. Kuppens S. Van Damme J. Van Petegem P. Lamote C. Boonen T. (2012). The role of teachers’ expectations in the association between children’s SES and performance in kindergarten: a moderated mediation analysis. *PLoS One* 7:e34502. 10.1371/journal.pone.0034502 22506023 PMC3323609

[B68] Stirrat M. Perrett D. I. (2010). Valid facial cues to cooperation and trust: male facial width and trustworthiness. *Psychol. Sci.* 21 349–354. 10.1177/0956797610362647 20424067

[B69] Strachan J. W. A. Kirkham A. J. Manssuer L. R. Over H. Tipper S. P. (2017). Incidental learning of trust from eye-gaze: effects of race and facial trustworthiness. *Vis. Cogn.* 25 802–814. 10.1080/13506285.2017.1338321

[B70] Strachan J. W. A. Kirkham A. J. Manssuer L. R. Tipper S. P. (2016). Incidental learning of trust: examining the role of emotion and visuomotor fluency. *J. Exp. Psychol.* 42 1759–1773. 10.1037/xlm0000270 27031325

[B71] Strachan J. W. A. Tipper S. P. (2017). Examining the durability of incidentally learned trust from gaze cues. *Q. J. Exp. Psychol.* 70 2060–2075. 10.1080/17470218.2016.1220609 27494048

[B72] Sussman A. B. Petkova K. Todorov A. (2013). Competence ratings in US predict presidential election outcomes in Bulgaria. *J. Exp. Soc. Psychol.* 49 771–775. 10.1016/j.jesp.2013.02.003

[B73] Terracciano A. Abdel-Khalek A. M. Ádám N. Adamovová L. Ahn C. K. Ahn H. N. (2005). National character does not reflect mean personality trait levels in 49 cultures. *Science* 310 96–100. 10.1126/science.1117199 16210536 PMC2775052

[B74] Thoits P. A. Hewitt L. N. (2001). Volunteer work and well-being. *J. Health Soc. Behav.* 42 115–131. 10.2307/3090173 11467248

[B75] Todorov A. Olson I. R. (2008). Robust learning of affective trait associations with faces when the hippocampus is damaged, but not when the amygdala and temporal pole are damaged. *Soc. Cogn. Affect. Neurosci.* 3 195–203. 10.1093/scan/nsn013 19015111 PMC2566771

[B76] Todorov A. Pakrashi M. Oosterhof N. N. (2009). Evaluating faces on trustworthiness after minimal time exposure. *Soc. Cogn.* 27 813–833. 10.1521/soco.2009.27.6.813

[B77] Tov W. Diener E. (2008). The well-being of nations: linking together trust, cooperation, and democracy. *Cooperation* eds Sullivan B. A. Snyder M. Sullivan J. L. (Malden, MA: Blackwell) 323–342.

[B78] van’t Wout M. Sanfey A. G. (2008). Friend or foe: the effect of implicit trustworthiness judgments in social decision-making. *Cognition* 108 796–803. 10.1016/j.cognition.2008.07.002 18721917

[B79] Xu F. Wu D. C. Toriyama R. Ma F. L. Itakura S. Lee K. (2012). Similarities and differences in Chinese and Caucasian adults’ use of facial cues for trustworthiness judgments. *PLoS One* 7:e34859. 10.1371/journal.pone.0034859 22514680 PMC3325928

[B80] Yu M. Saleem M. Gonzalez C. (2014). Developing trust: first impressions and experience. *J. Econ. Psychol.* 43 16–29. 10.1016/j.joep.2014.04.004

[B81] Zebrowitz L. Montepare J. (2005). Appearance DOES matter. *Science* 308 1565–1566. 10.1126/science.1114170 15947164

[B82] Zebrowitz L. Montepare J. (2008). Social psychological face perception: why appearance matters. *Soc. Pers. Psychol. Comp.* 2 1497–1517. 10.1111/j.1751-9004.2008.00109.x 20107613 PMC2811283

[B83] Zebrowitz L. Montepare J. Lee H. (1993). They don’t all look alike: individuated impressions of other racial groups. *J. Pers. Soc. Psychol.* 65 85–101. 10.1037//0022-3514.65.1.85 8355144

