# Corrigendum: Are Rich People Perceived as More Trustworthy? Perceived Socioeconomic Status Modulates Judgments of Trustworthiness and Trust Behavior Based on Facial Appearance

**DOI:** 10.3389/fpsyg.2019.02316

**Published:** 2019-10-11

**Authors:** Yue Qi, Qi Li, Feng Du

**Affiliations:** ^1^CAS Key Laboratory of Behavioral Science, Institute of Psychology, Chinese Academy of Sciences, Beijing, China; ^2^Department of Psychology, University of Chinese Academy of Sciences, Beijing, China

**Keywords:** socioeconomic status, facial judgment, trust, first impression, cooperation

There is an error in the Funding statement.

A correction has been made in the ***Funding*** statement:

“This research was supported by Beijing Natural Science Foundation (5184035); the National Natural Science Foundation of China (31470982, 31200782, and 31571161); the Scientific Foundation of the Institute of Psychology, Chinese Academy of Sciences (Y4CX033008); the Young Scientists Fund of the Institute of Psychology (Y5CX122005); and Magnetic Resonance Imaging Research Center, Institute of Psychology, CAS, and the China Scholarship Council.”

The authors apologize for this error and state that this does not change the scientific conclusions of the article in any way. The original article has been updated.

